# Quality of life of residents with dementia in long-term care settings in the Netherlands and Belgium: design of a longitudinal comparative study in traditional nursing homes and small-scale living facilities

**DOI:** 10.1186/1471-2318-11-20

**Published:** 2011-05-03

**Authors:** Alida HPM de Rooij, Katrien G Luijkx, Anja G Declercq, Jos MGA Schols

**Affiliations:** 1 Managing Director of De Kievitshorst Care Center, De Wever, Beneluxlaan 101, 5042 WN, Tilburg, The Netherlands; 2 Tilburg University, Tranzo Department, Tilburg, The Netherlands; 3 K.U. Leuven, Lucas, Leuven, Belgium; 4 Maastricht University/Caphri/Department of General Practice, Maastricht, The Netherlands

## Abstract

**Background:**

The increase in the number of people with dementia will lead to greater demand for residential care. Currently, large nursing homes are trying to transform their traditional care for residents with dementia to a more home-like approach, by developing small-scale living facilities. It is often assumed that small-scale living will improve the quality of life of residents with dementia. However, little scientific evidence is currently available to test this. The following research question is addressed in this study: Which (combination of) changes in elements affects (different dimensions of) the quality of life of elderly residents with dementia in long-term care settings over the course of one year?

**Methods/design:**

A longitudinal comparative study in traditional and small-scale long-term care settings, which follows a quasi-experimental design, will be carried out in Belgium and the Netherlands. To answer the research question, a model has been developed which incorporates relevant elements influencing quality of life in long-term care settings. Validated instruments will be used to evaluate the role of these elements, divided into environmental characteristics (country, type of ward, group size and nursing staff); basic personal characteristics (age, sex, cognitive decline, weight and activities of daily living); behavioural characteristics (behavioural problems and depression); behavioural interventions (use of restraints and use of psychotropic medication); and social interaction (social engagement and visiting frequency of relatives). The main outcome measure for residents in the model is quality of life. Data are collected at baseline, after six and twelve months, from residents living in either small-scale or traditional care settings.

**Discussion:**

The results of this study will provide an insight into the determinants of quality of life for people with dementia living in traditional and small-scale long-term care settings in Belgium and the Netherlands. Possible relevant strengths and weaknesses of the study are discussed in this article.

**Trial registration:**

ISRCTN: ISRCTN23772945

## Background

The substantial increase in the number of people with dementia worldwide implies that there will be much greater demand for both home care and residential care in the future [1]. For the Netherlands, the expectation is that in 2030 there will be 319,312 people with dementia, an increase of 65% over a period of 25 years [2]. The expectation for Belgium is that there will be 251,000 people aged over 65 with dementia in 2030, a rise of 56% over 25 years [3].

People usually prefer home care over residential care, but for a substantial number of people in the later stages of dementia staying at home is no longer possible [4]. In the last decade, the institutional regime in traditional, large nursing homes with a strongly medical and nursing-based approach, the hospital-like environment and the lack of privacy, has come in for heavy criticism in many countries [5-8]. To address this criticism, many large nursing home organizations are currently transforming their traditional care methods to a more home-like approach by developing small-scale living facilities [9-11]. The number of these small-scale living facilities has been increasing in many countries all over the world, such as Sweden, the United States, Germany, Belgium and the Netherlands. They take a variety of forms and the expansion is taking place at different rates in each country [5,12,13].

The assumption that small-scale living facilities will improve the quality of life of older persons with dementia compared to traditional nursing home care is often made by organizations that provide care for the elderly [8,14-16], as well as by politicians and other policymakers [17-20]. However, little scientific evidence is currently available about the effects of small-scale living on the quality of life of these residents [5,21]. Quality of life is defined by the World Health Organization (WHO) as "individuals' perceptions of their position in life in the context of the culture and value systems in which they live and in relation to their goals, expectations, standards and concerns" [22]. The physical, psychological, social and environmental domains are considered to be the most important indicators of quality of life [22,23].

The aim of this study is to contribute to the knowledge about the effects of small-scale living on the quality of life of residents with dementia by answering the following main research question: Which (combination of) changes in elements affects (different dimensions of) the quality of life of elderly residents with dementia in long-term care settings over the course of one year?

The relevant elements taken into account in this study are country (the Netherlands or Belgium), type of ward (traditional or small-scale), group size, nursing staff, age, sex, cognitive decline, weight, activities of daily living (ADL), behavioural problems, depression, use of restraints, use of psychotropic medication, social engagement and visiting frequency of relatives.

## Methods

This study is a longitudinal comparative study of elderly residents with dementia in long-term care settings in Belgium and the Netherlands.

### Sample size considerations

Sample size calculations are conducted for two groups (small-scale and traditional living) based on the primary outcome measure for residents, quality of life, as measured by the QUALIDEM [24]. Using an effect size of 0.50, a two-sided significance level α of 0.05 and a power of 80%, about 70 participants are needed in each group. Expecting an average drop-out rate of 20%, we aim to include about 180 residents. From the five long-term care settings that were selected for this study, 179 residents can be included.

### Small-scale living facilities and traditional care wards

Small-scale living facilities and traditional institutional psychogeriatric wards will be compared, based on the numerous differences between these two types of settings and the assumption that there will be a concomitant difference in the quality of life of the residents. Five long-term care settings have been selected for the study, of which two have traditional care wards and four have small-scale living wards: one of the settings has both small-scale and traditional wards. The long-term care settings were selected in advance, taking into account the comparability of the frailty of the residents. All residents willing to participate will be included in this study. An overview of the settings can be found in Table 1.

**Table 1:** Wards

Country	Long-term care setting	Small-scale wards N	Traditional wards N	Total at T0 N
*The Netherlands*	*A*	13	51	64

	*B*	24		24

	*C*	14		14

*Belgium*	*D*	47		47

	*E*		30	30

**Total N**		**98**	**81**	**179**

### Belgium and the Netherlands

The choice of these two countries was primarily based on the existing collaboration between Tilburg University (the Netherlands) and K.U. Leuven (Belgium). The use of data from two different countries affords the possibility of comparison and the opportunity to learn from each other. There are also advantages as regards data collection, in that the two countries are geographically adjacent and the spoken language in both countries is Dutch. However, there are also differences, for example in legislation and the funding of long-term care, the design and number of small-scale living facilities (more facilities in the Netherlands), the group size (groups are generally smaller in the Netherlands) and the speed of development (a more rapid expansion of small-scale living can be observed in the Netherlands) [8,25].

### Study population

The study will include elderly residents over 65 years of age, with dementia and who have been admitted to a long-term care setting.

Dementia is a complex syndrome, which manifests itself in various forms and is characterized by an initially gradual and progressively deteriorating impairment of the brain functions [26]. The DSM-IV employs the presence of multiple cognitive impediments with disorders in memory functions as a criterion for diagnosing the disease [27]. The memory disorders appear in varying combinations with changes in personality, mood and behaviour [26]. Residents with dementia are often legally incapable, and the law therefore stipulates that a legal representative be appointed to look after their interests. For this study, these representatives will be asked to give informed consent on behalf of the residents [28-30].

Owing to the cognitive decline, self-reporting is often no longer possible for people with dementia. Observation by one or more professional caregivers is therefore considered to be the best, most reliable and valid alternative method of data gathering [31]. Questionnaire sets validated for the target group have been selected for the study, which can be completed by a professional caregiver, namely a nurse or nursing assistant, who is familiar with the resident. One of the questionnaires is filled in by an independent psychologist interviewing the nurse or nursing assistant who is familiar with the resident, and one will be used to gather information from the residents themselves.

### Ethical approval and informed consent

The ethics committee at De Wever, Tilburg, gave its approval for the study in September 2008. The trial is registered as ISRCTN23772945. In practice, we will consider our ethical responsibility to be to the residents and family that we wish to include in our study. There will be virtually no inconvenience to the residents, because professional caregivers will fill in the questionnaires for them. The legal representatives of the residents will receive an information brochure containing information on all aspects of the research. An informed consent form will accompany the brochure, for the representative to sign and return. Only residents for whom such consent has been given will be included in the study. All those involved will be informed that they may end their participation in the study at any time. The privacy of the participating residents will be protected and all data will be analyzed anonymously.

### Conceptual model

**Figure 1:**
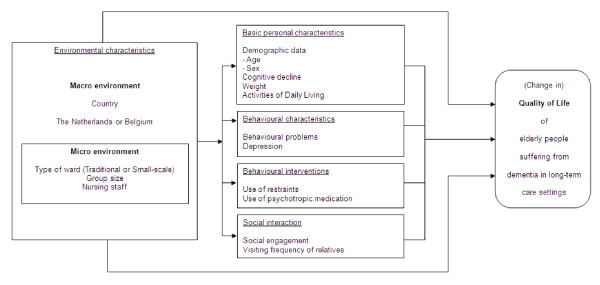
**Conceptual Model**.

The following conceptual model (see Figure 1) will be used to answer the research question. The model was developed after studying the most relevant factors affecting the quality of life of persons with dementia as reported in the literature [7,32-51]. The main outcome in this model is the quality of life of elderly residents with dementia in long-term care settings. The model shows two categories of influence on quality of life. The first category describes the environmental characteristics, divided into macro-environment (country) and micro-environment (type of ward, group size and nursing staff). The second category describes basic personal and behavioural characteristics, behavioural interventions and social interaction. We assume that the environmental characteristics influence quality of life both directly and indirectly through the personal, behavioural and social aspects. The elements of the conceptual model will be measured using valid and reliable instruments. All scales (except for the recording of country, type of setting, age, sex and staff formation) will be measured at three moments over the course of one year: T0 (baseline), T1 (after six months) and T2 (after 12 months). An overview of the measurements and records can be found in Table 2. Most of the scales and record forms will be filled in by a nurse or nursing assistant who is familiar with the resident. One of the scales is administered by interviewing a nurse or nursing assistant, and one will be used for gathering information from the resident by an independent psychologist. All characteristics, their relation to quality of life and the proposed instruments to be used in the study are discussed below.

**Table 2:** Measurements and records

	Measurement moment		
	**T0 (start)**	**T1 (6 months)**	**T2 (12 months)**

**Quality of life (QUALIDEM)**			

Positive self-image	X	X	X

Restless behaviour	X	X	X

Feeling at home	X	X	X

Caregiver relationship	X	X	X

Social relations	X	X	X

Social isolation	X	X	X

Negative affect	X	X	X

Positive affect	X	X	X

Having something to do	X	X	X

**Environmental characteristics**			

Country (The Netherlands or Belgium)	X		

Type of ward (traditional or small-scale)	X		

Group size	X		

Nursing staff	X		

**Basic personal characteristics**			

Demographic data			

- Age	X		

- Sex	X		

Cognitive decline (S-MMSE)	X	X	X

Weight (Dossier)	X	X	X

Activities of daily living (Barthel Index)	X	X	X

**Behavioural characteristics**			

Behavioural problems (NPI-NH)	X	X	X

Depression (CSDD)	X	X	X

**Behavioural interventions**			

Use of restraints (Dossier)	X	X	X

Use of psychotropic medication (Dossier)	X	X	X

**Social interaction**			

Social Engagement (RISE from RAI)	X	X	X

Visiting frequency of relatives (Questionnaire relatives)	X	X	X

### Quality of life

Quality of life is a broad concept. The WHO originally divided quality of life into six domains incorporating physical health, psychological state, level of independence, social relationships, personal beliefs and the relationships to features of the environment [23]. Empirical evidence from the WHO showed that a four-domain solution might fit the data better in both ill and well populations:

1. The physical domain (pain and discomfort; energy and fatigue; sleep and rest);

2. The psychological domain (positive affect; cognition, memory and concentration; self-image; appearance; negative affect);

3. Social relations domain (personal relationships; social support; sexual activity);

4. Environmental domain (safety; financial resources; availability and quality of health care; access to new information and skills; leisure time; mobility) [22,23].

Qualidem (Quality of life in dementia) is a measurement scale used to determine aspects of the quality of life of elderly residents with dementia [24]. Using this scale, it is possible to determine how residents deal with and experience their immediate environment and what kind of relationship the elderly resident has with care giving staff and other residents. This instrument was developed and validated [52] specifically for residents in long-term care settings, over the age of 65 years and suffering from mild to severe dementia. Two professional caregivers, a nurse or nursing assistant who are familiar with the resident, observe the elderly resident and complete the questionnaire together. In this way, the scale provides a quality of life profile of residents with dementia [53]. The questionnaire comprises 40 items that can be divided into nine subscales. The subscales need to be assessed individually and are not suitable for calculating a total score. The subscales are Caregiver relation, Positive affect, Negative affect, Restless behaviour, Positive self-image, Social relations, Social isolation, Feeling at home and Having something to do. For each item, an answer between '0 = never' and '3 = often' can be chosen. Administering the questionnaire generally takes around 15 minutes [24].

### Environmental characteristics

The relevance of country and type of ward for quality of life has already been discussed. Group size is an important factor from the micro-environment, because wards can vary in the number of residents (between six and 27), and nursing staff is an important factor, because staffing levels and education level can differ. At T0 country (the Netherlands or Belgium), type of ward (small-scale or traditional), group size (between six and 27) and nursing staff (staffing levels of qualified nurses and of other assistant staff) are recorded.

### Basic personal characteristics

#### Demographic data

As regards the basic personal data of the residents, their age and sex will be recorded at T0.

#### Cognitive decline

To provide an indication of the level of cognitive impairment, cognitive decline is included in the conceptual model. Cognitive decline has been shown to correlate with factors that are important for the quality of life, such as the occurrence and severity of problematic behaviours [33] and activities of daily living [34].

The Standardized Mini Mental State Examination (S-MMSE) measures the cognitive decline of the elderly person as the dementia progresses. In order to diagnose the severity of the influence of the disease on cognition and motor skills, the elderly person with dementia is asked to answer a series of 11 questions. For example, the resident is asked where he or she is, why they are there, what day of the month it is, to remember some words, write a sentence, copy a drawing and perform a few tasks (closing eyes/folding paper). The maximum possible test score is 30 [54]. The scale has been validated using psychometric analyses, with the conclusion that the S-MMSE is a reliable instrument and a valuable tool for assessing cognitive function [55]. The scale has a cut-off value of 27 for highly educated persons and 24 for lower-educated individuals [56].

#### Weight

Body weight can be considered as a general measure of health status. It is known that many persons with dementia begin to lose weight shortly after the onset of the disease due to a variety of physiological, psychological, medical and environmental factors [57,58]. Moreover, the literature shows that changes in the environment may improve nutritional intake [57]. This factor is therefore considered to be of relevance for this study and the most recent weight of the resident in kilograms, fully clothed and with shoes on, is taken from the personal records of the resident.

#### Activities of Daily Living (ADL)

Activities of daily living (ADL) may be considered as an indication of functional status. The literature shows that fostering ADL independence has a positive effect on the quality of life of persons with dementia [35,36].

ADL can be measured using the Barthel Index, which includes ten basic activities of daily living, namely personal hygiene, using the toilet, getting dressed, walking up and down stairs, bathing, mobility, (in)continence, requiring assistance in transferring from bed to chair, and requiring assistance with feeding. The scale is completed by a nurse or nursing assistant working in the facility who is familiar with the resident, and takes about five minutes. A score of 0 to 2 or 0 to 3 is recorded for each activity. The maximum possible score is 20. A score of 0 to 4 = Completely dependent on others; 5 to 9 = Requires lots of help; 10 to14 = Needs help but can do things alone, 15 to 19 = Reasonably independent, and 20 = Completely ADL-independent [59]. The Barthel Index is a valid and reliable measure [60].

### Behavioural characteristics

#### Behavioural problems

Behavioural problems, such as delusions, hallucinations, agitation, depression, anxiety, elation, apathy, disinhibition, irritability, aberrant motor behaviour, sleep problems and eating disorders are frequently seen in elderly people with dementia. Studies report that on average, mild to severe behavioural problems occur in 64% of cases of dementia [39], and that between 80% [40] and 90% [41] of all elderly people with dementia will develop at least one symptom of behavioural problems during the entire course of their disease. These problems cause distress and influence the quality of life not just of the elderly person concerned, but also of their family and professional caregivers [42].

The Neuropsychiatric Inventory - Nursing Home version (NPI-NH) questionnaire can be used by an independent psychologist to interview a nurse or nursing assistant working in the facility, who is familiar with the resident, about possible neuropsychiatric symptoms from which an elderly person is suffering. These symptoms include delusions, hallucinations, agitation/aggression, phobia, uninhibited behaviour, depression/dysphoria, euphoria, apathy/indifference, neuroticism, aimless repetitive behaviour, eating disorders and sleeping disorders. The NPI-NH gives an insight into the severity (1 to 3), frequency (1 to 4) and workload (0 to 5) of each of the separate behavioural disorders [61]. A total NPI-NH score can be calculated by adding together all twelve component scores (which are the product of the frequency and severity scores) [62]. Administering the questionnaire generally takes about 15 minutes. The psychometric properties and factor structure of the NPI-NH have been assessed, showing internal consistency, reliability, convergent validity and discriminant validity [63].

#### Depression

People with dementia often suffer from depressive symptoms, such as sadness, lack of energy, low reactivity to pleasant events and multiple physical complaints [43,44]. These depressive symptoms have been shown to correlate negatively with quality of life [45].

The Cornell Scale for Depression in Dementia (CSDD) was developed specifically for identifying depressive symptoms in elderly people with dementia [43]. The CSDD incorporates mood, behavioural disorders, physical characteristics of depression and cyclical functions and disorders in cognitive content. A nurse observes the elderly person and fills in an observational scale (containing 19 items and ranging from: a = cannot be judged, 0 = absent, 1 = mild, 2 = severe). The item scores are added together. Scores above 10 indicate a probable major depression. Scores above 18 indicate a definite major depression. Scores below six are generally associated with absence of significant depressive symptoms [43]. The CSDD has been assessed as a valid screening tool for depression in the elderly, being equally valid in populations with and without dementia [64].

### Behavioural interventions

#### Use of restraints

The use of restraints, including physical restraints (belts), is common practice in the long-term care setting [46,47]. Since these measures are not always effective and have other known negative physical, psychological and social consequences, their usage will influence quality of life [46,47]. Therefore, the number and type of restraints used by the resident are recorded. The range of restrictive measures includes fixation with belts, such as a large bed belt, a small bed belt, a fastening belt in a chair or wheelchair, securing the person to the bed with a blanket, using an adjustable tabletop in chair, restraining of limbs and the use of bedrails, but also the application of technological restraints such as movement detection mats or movement detection sensors in the bedroom.

#### Use of psychotropic medication

The use of psychotropic medication is common among residents with dementia in long-term care settings. It is known that the use of this type of medication may have a detrimental effect on quality of life [48-50]. The use and number of sedatives, antidepressants and antipsychotics is taken from the actual medication file in the personal notes of the resident.

### Social interaction

#### Social engagement

Participation in joint activities, such as drinking coffee together, active or passive participation in a game or taking a walk together, is related to a higher quality of life [51], while activities such as interacting with a pet and making music as part of a group have been shown to provide positive cognitive stimulation in persons with dementia [6].

The Revised Index for Social Engagement (RISE) measures the social involvement of elderly residents suffering from dementia with other residents, professional caregivers and relatives. The scale contains eight questions about the social interaction of the resident. A nurse or nursing assistant who is familiar with the resident completes the questions by marking whether or not the specific social situation mentioned in the question has occurred over the last seven days. Completing the questionnaire takes about five minutes. RISE is one of the scales derived from the larger instrument Resident Assessment Instrument 2.0 (RAI 2.0) [65]. The RAI 2.0 is used to assess a variety of factors related to the functioning of elderly residents in care homes [66]. The reliability and validity of the RISE has been assessed individually and it was found to be a valuable and stable measure for assessing social engagement in nursing homes [67], including patients with cognitive impairments [65]. The scale does not have an established cut-off value.

#### Visiting frequency of relatives

The relationship with relatives is important for residents with dementia, because this relationship may have a positive influence on the behavioural and psychological symptoms of the disease [68,69]. A nurse or nursing assistant who is familiar with the resident will record whether the resident has visitors: almost every day, once or twice a week, once every two weeks, once a month or less than once a month.

## Analyses

Comparisons will be made in the analysis to explore whether there are differences in the combination of elements that influence the quality of life of residents living in a long-term traditional or small-scale setting in the Netherlands and in Belgium.

**Figure 2:**
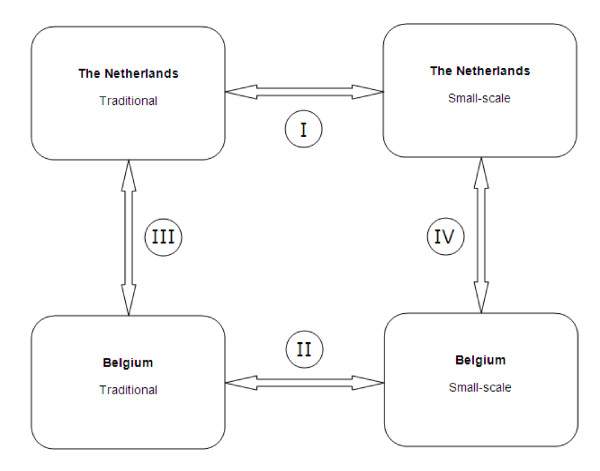
**Comparisons**.

To enable comparison of quality of life in the two countries and in both types of wards, the comparative analyses shown in Figure 2 will be central. The two types of settings will be compared within each country. Additionally, the Dutch small-scale living facilities will be compared with their Belgian counterparts. The same comparison will be made for the traditional wards in both countries.

To make a comparison, the data will be analyzed using descriptive, quantitative and qualitative analyses. The quantitative analyses will include cross-sectional and longitudinal analyses using SAS 9.2.1^©^. The null hypothesis that 'the patterns of change in the mean response over time on the Qualidem subscales are the same in both types of long-term care settings' will be tested. The qualitative analyses will be carried out using Qualitative Comparative Analysis using TOSMANA^© ^[70]. One of the general advantages of QCA is that it combines the strength of qualitative research (within-case knowledge) with the strength of quantitative enquiry (cross-case comparison) [71]. The most conventional and intuitive type of QCA analysis will be employed for the analyses: crisp-set Qualitative Comparative Analysis (csQCA). In csQCA, a dichotomous data table will be built, based on within-case knowledge, and from this dichotomous data table a set of necessary and sufficient conditions leading to a certain outcome will be deduced [72].

This approach integrates key strengths of both qualitative (case-oriented) and quantitative (variable-oriented) methods and allows triangulation. It provides a double check to ensure that valid results are obtained despite the relatively small number of participants.

## Discussion

This study will provide an insight into determinants of quality of life for people with dementia living in traditional and small-scale long-term care settings in Belgium and the Netherlands. Due to ethical considerations, a randomised controlled trial is impossible. Although they have been selected based on their similar view of care giving, the small-scale and traditional wards in the two countries are not entirely comparable. This could potentially influence the results of the study. There are differences in legislation as well as in the organization and implementation of residential care between both countries.

Moreover, the settings are real-life care settings and have specific characteristics that may vary within and between countries. The analyses will therefore be controlled for relevant differences between and within countries, such as group size, differences in the length of existence of the small-scale living facilities (ranging from September 2006 to December 2007) and differences in nursing staff, and for significant variations in the basic data of the residents.

Due to the age and frailty of the participants and the fact that the study will be conducted over the course of an entire year, there will be drop-outs, mainly due to death or occasionally to residents being moved to another institution.

Despite these limitations, collecting data from residents on various different elements, in different countries and different types of ward provides added value, because of the learning aspects and because it may enable certain patterns in the data to be discerned more clearly.

## Competing interests

The authors declare that they have no competing interests.

## Authors' contributions

AHPMR, KGL, AGD and JMGAS are involved in the study design, and critically reviewed and approved the final manuscript. AHPMR drafted the manuscript.

## Pre-publication history

The pre-publication history for this paper can be accessed here:

http://www.biomedcentral.com/1471-2318/11/20/prepub
